# Kaempferol Inhibits Pancreatic Cancer Cell Growth and Migration through the Blockade of EGFR-Related Pathway *In Vitro*

**DOI:** 10.1371/journal.pone.0155264

**Published:** 2016-05-13

**Authors:** Jungwhoi Lee, Jae Hoon Kim

**Affiliations:** 1 Faculty of Biotechnology, College of Applied Life Science, SARI, Jeju National University, Jeju, Republic of Korea; 2 Subtropical Horticulture Research Institute, Jeju National University, Jeju, Republic of Korea; 3 Subtropical/tropical organism gene bank, Jeju National University, Jeju, Republic of Korea; University of Verona, ITALY

## Abstract

Pancreatic cancer is one of the most appalling cancers with a pessimistic prognosis. Despite many therapies, there has been no improvement of survival rates. In this study, we assessed the anti-cancer effects of kaempferol, a well known flavonoid having functional bio-activity against various malignant tumors. Kaempferol had anti-cancer effects on Miapaca-2, Panc-1, and SNU-213 human pancreatic cancer cells. In a dose-dependent manner, kaempferol decreased viability of these pancreatic cancer cells by increasing apoptosis. In particular, kaempferol effectively inhibited the migratory activity of human pancreatic cancer cells at relatively low dosages without any toxicity. The anti-cancer effect of kaempferol was mediated by inhibition of EGFR related Src, ERK1/2, and AKT pathways. These results collectively indicate that kaempferol, a phytochemical ingredient reported to have anti-viability and anti-oxidant properties, can act as a safety anti-migration reagent in human pancreatic cancer cells, which provide the rationale for further investigation of kaempferol as a strong candidate for the potential clinical trial of malignant pancreatic cancers.

## Introduction

Pancreatic cancer is a fatal disease. The 5-year survival rate of patients with pancreatic cancer is below 1% [1]. One of the major features of pancreatic cancer is the early propagation and progression of local tumors. These features cause serious complications in patients with pancreatic cancer. Therefore, most patients have an untimely death [2]. The metastatic tendency of tumor cells is a major obstacle in the treatment of pancreatic cancer. However, researchers have not been to elucidate how malignant pancreatic cancer cells migrate from a primary tumor site to a distant organ [3]. Furthermore, with the presently available anti-cancer drugs and radiation therapy have an inconsequential impact on most patients with pancreatic cancer. Therefore, extensive studies must be conducted to explore the molecular mechanisms responsible for these characteristics and develop additional strategies to improve the treatment outcomes in patients with pancreatic cancer.

Flavonoids are polyphenolic compounds that are abundantly found in various plants [4]. They provide several health benefits due to their anti-oxidant and anti-inflammation activities. Previous studies have reported that flavonoids inhibit various stages of cancer, such as proliferation and angiogenesis [5]. In particular, kaempferol, a well-known flavonoid, has remarkable bio-activity against various malignant tumors. Kaempferol is abundantly found in tea, apples, strawberries, beans, and citrus fruits [6]. Kaempferol regulates various features of cancer cells, such as apoptosis [7–9], cell cycle [10,11], and inflammation [12,13]. In addition, kaempferol also inhibits the migration and invasion activity of medulloblastoma and breast cancer cells [14,15]. But its’ anti-migratory effect on pancreatic cancer cells is unknown.

Epidermal growth factor receptor (EGFR) is a receptor tyrosine kinase that is particularly activated when interacted between EGFR and growth factors of the EGF-family. Several mechanisms lead to the abnormal activation of EGFR observed in various cancers, *i*.*e*., EGFR over-expression, mutation, and ligand-receptor independent stimulation. EGFR is the representative receptor underlying various tumor phenotypes, such as proliferation, anti-apoptotic advantage, migration, and invasion in malignant tumors including pancreatic cancers [16–19]. Upregulated EGFR is also found in pancreatic cancer tumors, which correlate with a dismal prognosis [20].

In this study, we investigated the nature of kaempferol’s anti-cancer impact on human pancreatic cancer cell lines through the blockade of EGFR-related intracellular signaling. Our results indicate that kaempferol may be a safe inhibitor against malignant pancreatic cancers.

## Materials and Methods

### Cell culture and reagents

**Fig 1:**
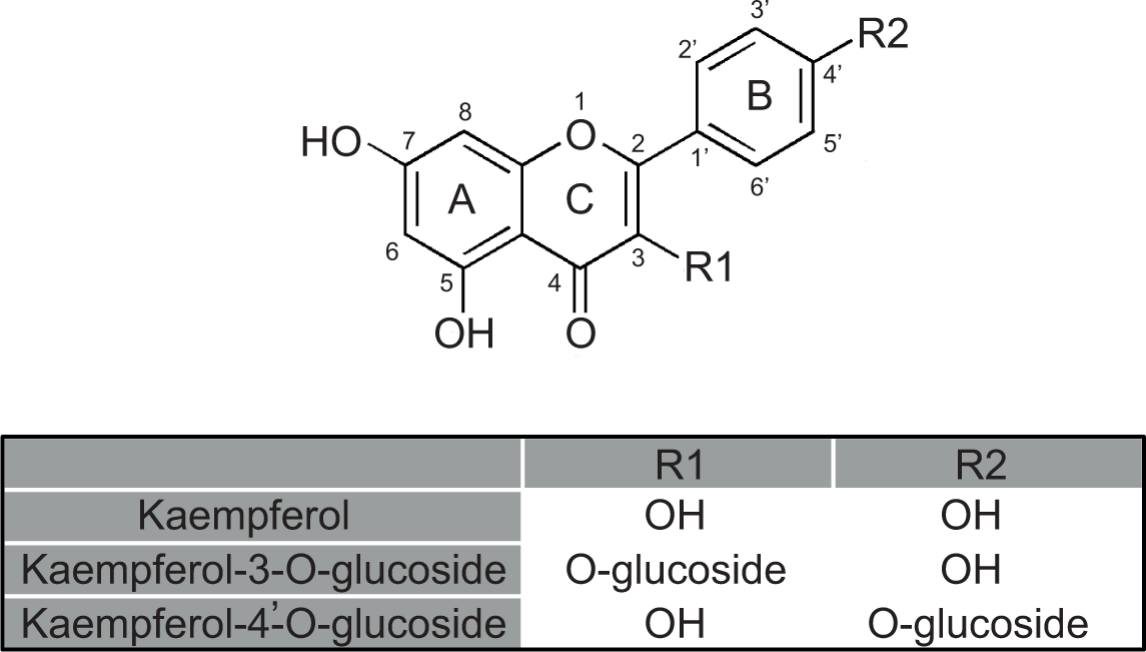
Structure of kaempferol and its derivatives.

Miapaca-2, Panc-1, and SNU-213 human pancreatic cancer cell lines were obtained from the Korean Cell Line Bank (KCLB, Seoul, Korea). The cells were grown either in DMEM (Miapaca-2 and Panc-1) or RPMI-1640 (SNU-213) medium supplemented with 10% fetal bovine serum (Gibco-BRL, Gaithersburg, MD, USA), 1×10^5^ unit/L Penicillin, and100 mg/L Streptomycin (Invitrogen, Carlsbad, CA, USA) at 37°C. The cell culture was incubated in a humidified atmosphere containing 5% CO_2_ as previously described [21]_._ Human umbilical vein endothelial cells (HUVECs) were purchased from the American Type Culture Collection (ATCC, Manassa, VA, USA). HUVECs were grown in EGM-2 Bulletkit medium (Lonza Biologics, Hopkinton, MA) at 37°C in a humidified atmosphere containing 5% CO2. All experiments were performed using HUVECs within 3–6 passages. EGFR inhibitor (Gefitinib) and pan caspase inhibitor (Z-VAD-FMK): these inhibitors were purchased from Santa Cruz Biotechnology (Santa Cruz, CA, USA). The antibodies for phospho-EGFR (Tyr1068), EGFR, phospho-Src (Tyr416), Src, phospho-AKT (Ser473), AKT, phospho-extracellular signal-regulated kinase (ERK)1/2 (Thr202/Tyr204), ERK, proliferating cell nuclear antigen (PCNA), and glyceraldehyde-3-phosphate dehydrogenase (GAPDH) were obtained from Cell Signaling Technology (Beverly, MA, USA). Kampferol, kampferol-3-O-glucoside, and kampferol-4’-O-glucoside (Fig 1) were purchased from Sigma-Aldrich (St. Louis, MO, USA).

### Measurement of cell viability

Cell viability was determined using a WST-1 solution: 2-(4-iodophenyl)-3-(4-nitrophenyl)-5-(2,4-disulfophenyl)-2H-tetrazolium solution (Boehringer Mannheim, Mannheim, Germany). In this assay, Miapaca-2, Panc-1, and SNU-213 cells (5 × 10^3^/well) were seeded in 96-well plates (Nunc, Roskilde, Denmark). The cells were maintained in a culture medium for 24 h. During this period, they were treated with various dosages of kaempferol. Cells were incubated at 37°C for an additional 72 h. Ten microliters of WST-1 solution was added to each well. After a 10-min incubation at room temperature, the absorbance was measured at 450 nm using a microplate reader (Bio-Rad, Richmond, CA, USA).

### Migration assay

Cell migration assays were performed using Transwell Permeable Supports (Corning Costar, Lowell, MA) having a pore size of 8.0 μm. Polycarbonate filters were pre-coated with fibronectin (Sigma-Aldrich, St. Louis, MO) using a medium containing 10 mg/L of phosphate-buffered saline; the pre-coating process was carried out for 30 min at room temperature. The lower chamber was filled with 500 μl of RPMI-1640 medium containing 10% serum. After subjecting the cells to a 15 h starvation in serum free RPMI-1640 medium, we suspended the cells (5 × 10^4^ cells/well) in 100 μl serum-free medium and loaded into every upper chamber. After keeping these cells in the aforementioned medium for 6 h at 37°C, we removed the cells on the upper surface of the filter using a cotton swab. Then, we fixed and stained the filters using 1% crystal violet solution. The eluted dye was measured at 560 nm in an ELISA reader (Bio-Rad).

### Invasion assay

Invasion assays were performed using growth factor-reduced Matrigel (BD Biosciences, San Diego, CA, USA) coated on 24-Transwell plates according to the supplier’s protocol. Cells were applied to the upper-chamber containing RPMI without serum for 20 h, and cells that had migrated to the back side of the filter after 24 h were stained. The eluted dye was measured at 560 nm in an ELISA reader.

### Kaempferol target selection

For selecting the targets of kaempferol, we used the traditional Chinese medicine systems pharmacology database and analysis platform (TCMSP) as previously described [22].

### Western blot analysis

For evaluating phosphoylation process in various molecules, Western blotting was performed as previously reported [23]. In this method, we treated cultured Miapaca-2 and Panc-1 cells with kaempferol for indicated fixed number of times. Thereafter, we washed these cells with pre-chilled phosphate buffered saline (PBS). For whole cell lysates, cells were lysed in M-PER lysis buffer (Thermo Scientific, Bonn, Germany) containing protease and phosphatase inhibitors. The total quantity of protein was determined by the BCA quantification method (Bio-Rad). Cell lysates were separated by 10% SDS-PAGE and transferred into nitrocellulose membrane (Amersham Bioscience, Buckinghamshire, UK). These membranes were blocked using 5% bovine serum albumin and 2% Tween in Tris buffered saline. We diluted the antibodies specific for phospho-EGFR (Tyr1068), EGFR, phospho-Src (Tyr416), Src, phospho-AKT (Ser473), AKT, phospho-ERK1/2 (Thr202/Tyr204), ERK, and GAPDH in a blocking buffer at 1:1000 ratio. Thereafter, we incubated these antibodies overnight at 4°C. To visualize bands in an X-ray film, we used horseradish peroxidase-conjugated donkey anti-rabbit or donkey anti-mouse secondary antibody (Santa Cruz, CA, USA) along with Western blot detection reagent (iNtRON, Seoul, Korea). The bands were measured by densitometry analysis that was performed using ImageJ analysis software.

### Statistical analyses

Data were presented as the mean ± standard deviation (SD). The Student’s *t*-test was used to determine the levels of significance, which were then used for comparing two independent samples. The groups were compared by one-way analysis of variance (ANOVA). In this case, Tukey’s *post-hoc* test was applied to significant main effects (SPSS 12.0K for Windows; SPSS Inc., Chicago, IL, USA).

## Results

### Kaempferol inhibits human pancreatic cell viability through the apoptosis pathway

Kaempferol at approximately 10 μM induces significant anti-viability effect in pancreatic cancer cells through the apoptosis [9]. To ascertain the anti-cancer effect of kaempferol and its derivatives, Miapaca-2, Panc-1, and SNU-213 pancreatic cancer cells were incubated in various concentrations of kaempferol, kaempferol-3-O-glucoside, and kaempferol-4’-O-glucoside (0, 10, 25, and 50 μM). Compared with the control cells that were treated with PBS, 50 μM of kaempferol specifically inhibited the cell viability by approximately 40% in Miapaca-2 cells, 15% in Panc-1 cells, and 10% in SNU-213 cells. In contrast, kaempferol-3-O glucoside and kaempferol-4’-O glucoside did not have any inhibitory effect on Miapaca-2, Panc-1, and SNU-213 cells under identical conditions (S1 Fig).

**Fig 2:**
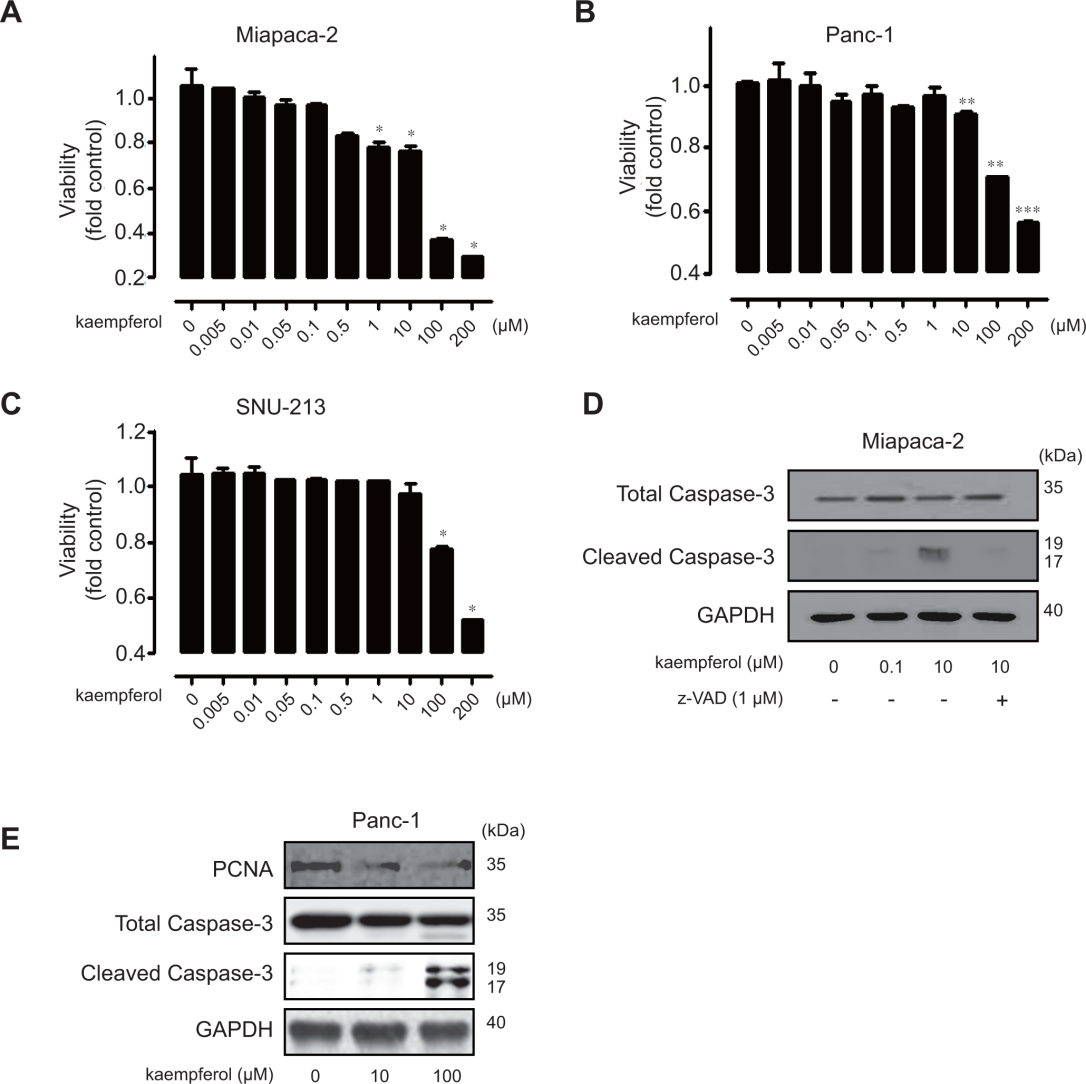
The effects of different doses of kaempferol on viability of human pancreatic cancer cells. (A-C) Miapaca-2, Panc-1, and SNU-213 human pancreatic cancer cells were incubated with varying doses of kaempferol for 72 h. The viability was measured by WST-1 assay (Data represent the percentage ± SD and are representative of three individual experiments, *p < 0.05, **p < 0.01, ***p < 0.001). (D) Miapaca-2 cells were incubated either in the absence or presence of kaempferol (0, 0.1, and 10 μM) and/or z-VAD (1 μM) for 72 h. Cell lysates were subjected to immunoblot analysis using antibodies against total caspase-3, cleaved-caspase-3, and GAPDH. (E) Panc-1 cells were incubated with different dosages of kaempferol (0, 10, and 100 μM) for 72 h. Cell lysates were subjected to immunoblot analysis using antibodies against PCNA, total caspase-3, cleaved-caspase-3, and GAPDH.

To further investigate the specific anti-cancer effect of kaempferol, Miapaca-2, Panc-1, and SNU-213 cells were incubated in various concentrations of kaempferol (0, 0.005, 0.01, 0.05, 0.1, 0.5, 1, 10, 100, and 200 μM). Exposure to kaempferol caused no significant alteration in cell viability provided it was present in relatively low dosages: up to 0.5 μM in Miapaca-2, up to 1 μM in Panc-1, and up to 10 μM in SNU-213 cells. However, higher dosages of kaempferol had a significant inhibitory effect on cell viability. Compared with the control cells that were treated with PBS, 200 μM kaempferol inhibited the cell viability by approximately 70% in Miapaca-2 cells, 45% in Panc-1 cells, and 50% in SNU-213 cells (Fig 2A, 2B and 2C).

To ascertain the anti-viability effect of kaempferol, we further investigated the caspase-3-mediated apoptosis in Miapaca-2 pancreatic cancer cells that were most sensitive to kaempferol treatment. We initially certified that 10 μM kaempferol significantly reduced cell viability, which was significantly suppressed by pretreatment of the pan-caspase inhibitor, z-VAD (data not shown). In addition, kaempferol induced the cleavage of caspase-3, which was effectively suppressed when Miapaca-2 cells were pre-treated with a z-VAD (Fig 2D). Similar results were obtained in 100 μM kaempferol treated Panc-1 and SNU-213 cells (Fig 2E and data not shown). The abundance of PCNA was also decreased during apoptosis as previously reported [24].

These results indicated that kaempferol can effectively inhibit the growth of pancreatic cancer cells provided it is administered in appropriate doses; it induces *in vitro* apoptosis of pancreatic cancer cells.

### Kaempferol inhibits migration of human pancreatic cancer cells even at low doses

**Fig 3:**
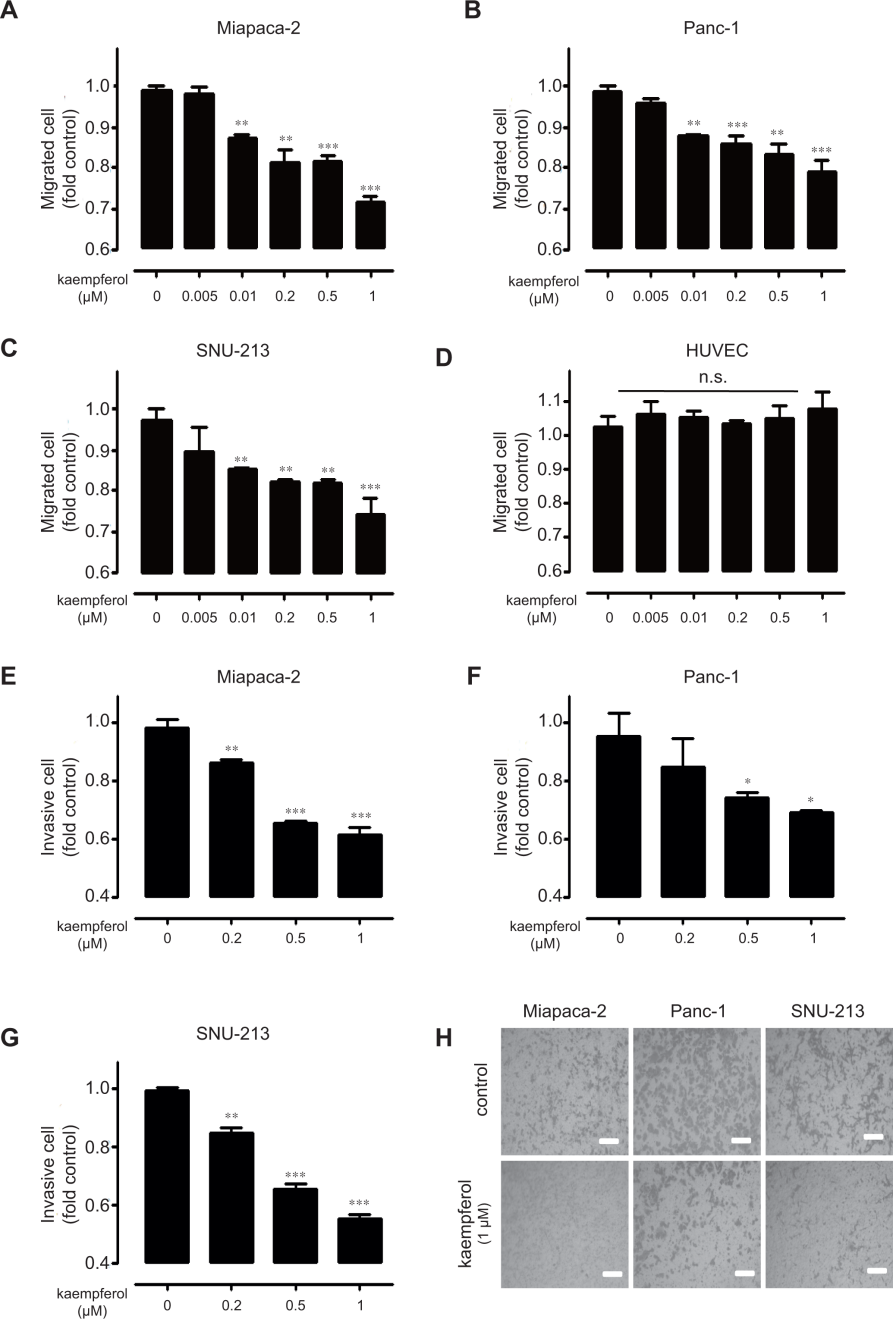
The effect of different doses of kaempferol on migration of human pancreatic cancer cells. (A-D) Miapaca-2, Panc-1, SNU-213 cells, and HUVECs were incubated with varying doses of kaempferol for 6 h. The migration activities were evaluated using the transwell-migration assay (Data represent the percentage ± SD and are representative of three individual experiments, n.s. means non-significant, *p < 0.05, **p < 0.01, ***p < 0.001). (E-G) Miapaca-2, Panc-1, and SNU-213 cells were incubated with varying doses of kaempferol for 20 h. The invasive activities were evaluated using the Transwell-invasion assay assay (data represent the percentage ± SD and are representative of three individual experiments, *p < 0.05, **p < 0.01, ***p < 0.001). (H) Representative image of Trans-well invasion assay (scale bar = 50 μm).

To further verify the anti-cancer effect of kaempferol, we investigated the inhibitory effect of kaempferol on migration of Miapaca-2, Panc-1, and SNU-213 cells. Compared with the control cells treated with PBS, serum-induced migration of Miapaca-2 cells was decreased up to approximately 30% after they were treated with 1 μM kaempferol (Fig 3A). After being treated with 1 μM kaempferol under the same conditions, migration of Panc-1 and SNU-213 cells were inhibited up to approximately 20% and 30%, respectively (Fig 3B and 3C). Treatment with up to 1μM kaempferol-3-O-glucoside and kaempferol-4’-O-glucoside had no effects on migration of Miapaca-2, Panc-1, and SNU-213 cells (S2 Fig). Next, we investigated the basal toxicity of kaempferol using HUVECs as representative normal cells. Kaempferol treatment (0, 0.005, 0.01, 0.2, 0.5, and 1 μM) did not affect migration and viability of HUVECs (Fig 3D and data not shown). In addition, we assessed invasive activities following the dose-dependent treatment of kaempferol in Miapaca-2, Panc-1, and SNU-213 cells. Compared with the control cells treated with PBS, serum-induced invasion of Miapaca-2, Panc-1, and SNU-213 cells was inhibited up to 40%, 30%, and 40%, respectively, after they were treated with 1 μM kaempferol (Fig 3E, 3F and 3G). Fig 3H shows the representative images of anti-invasion effect produced by 1 μM kaempferol treatment. Kaempferol specifically inhibited the serum-induced migration and invasion of human pancreatic cancer cells at a relatively low dosage without any toxicity.

### Kaempferol has anti-cancer effects through EGFR-related signaling pathway in human pancreatic cancer cells

**Fig 4:**
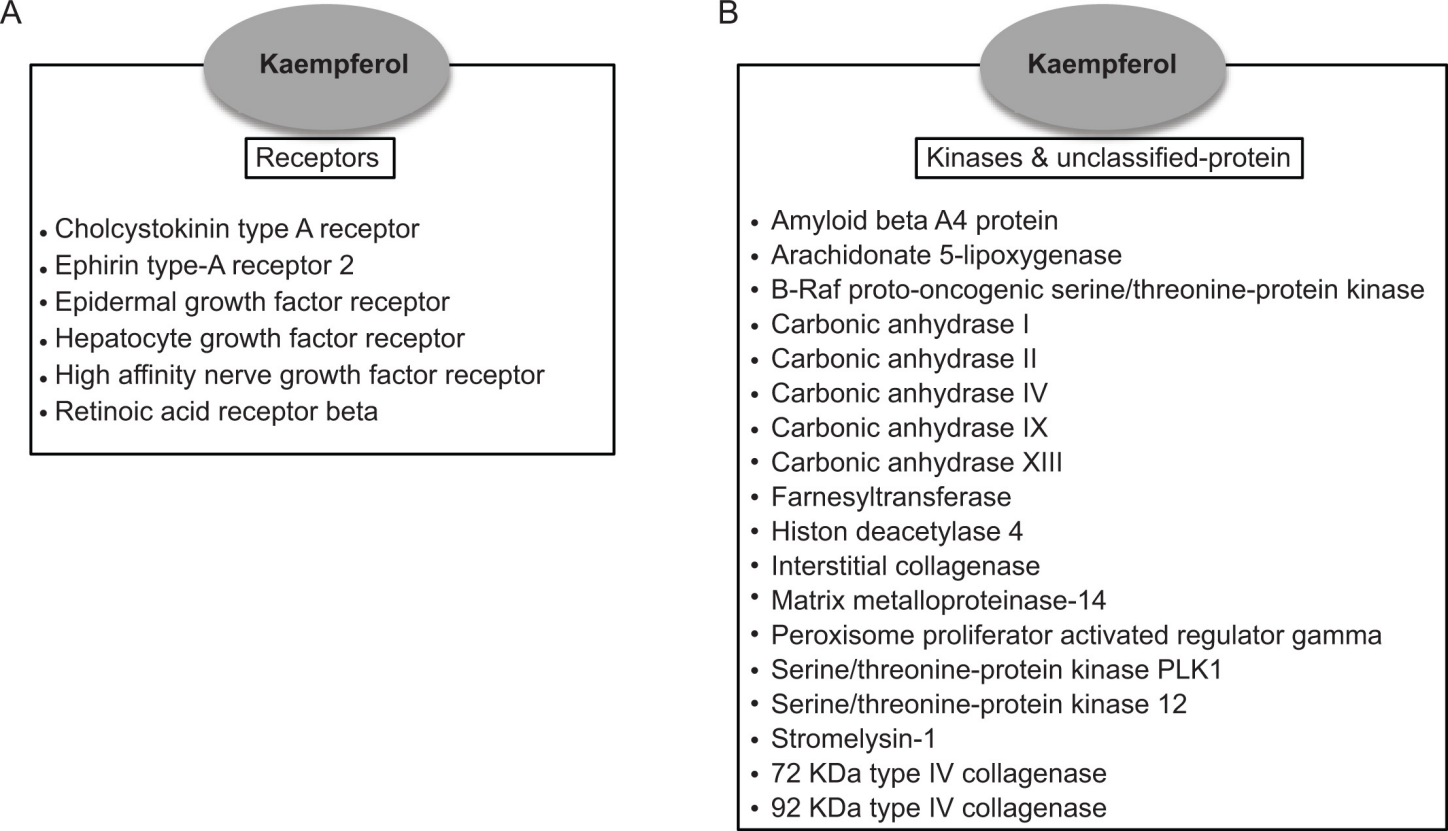
Potential targets of kaempferol in pancreatic cancers. Twenty-four potential targets using the TCMSP database were subdivided into receptor (A) and kinases/unclassified-protein (B) as their functions.

Various targets could be presupposed according to chemicals from herbal medicines using the public TCMSP database [22]. We selected 24 pancreatic cancer cell targets of kaempferol using the TCMSP database; they included six receptors including epidermal growth factor receptor (EGFR), 16 kinases, and two unclassified-proteins (Fig 4A and 4B).

**Fig 5:**
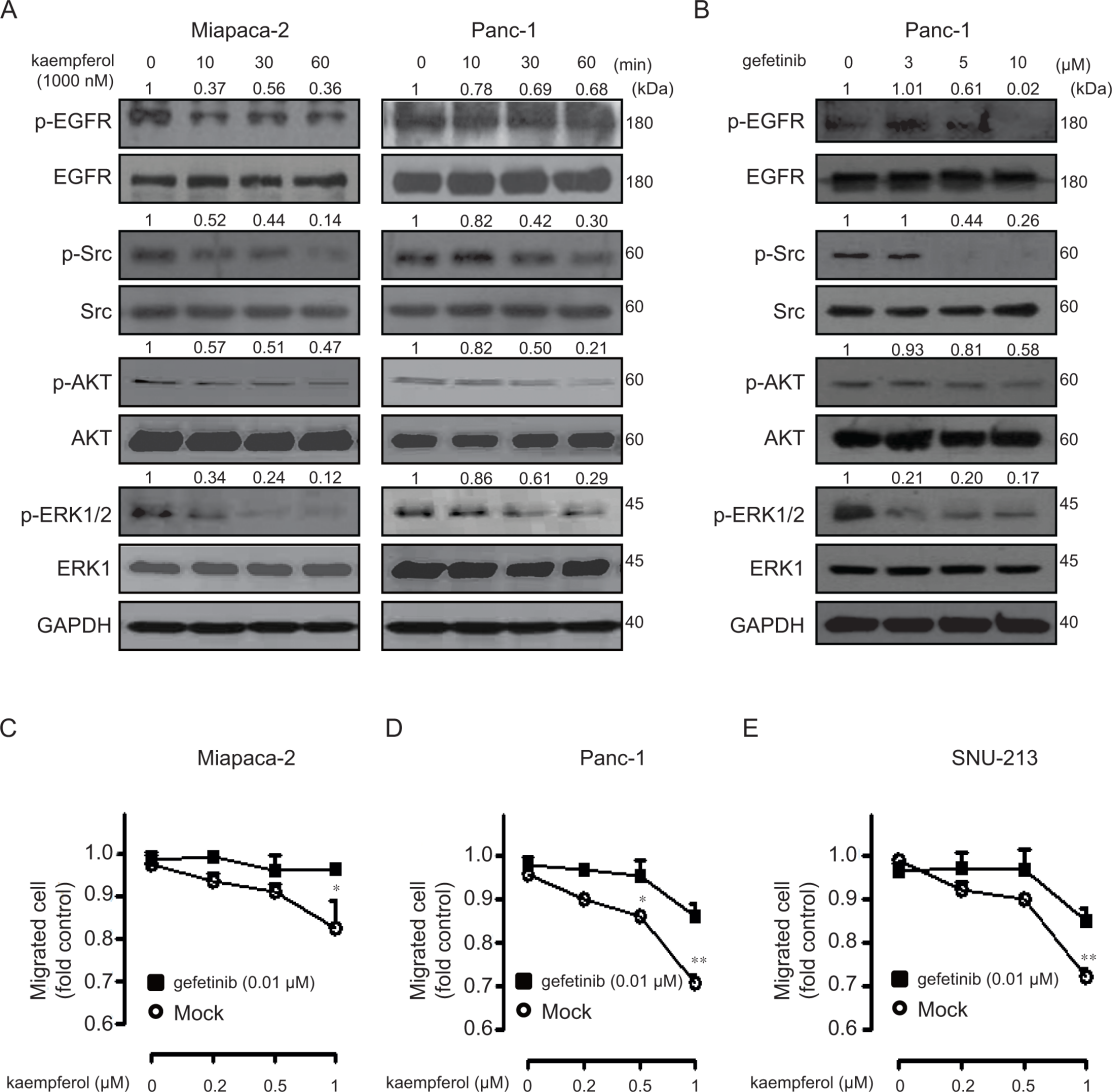
Intracellular signaling by a time-dependent kaempferol in human pancreatic cancer cells. (A) Miapaca-2 and Panc-1 cells were incubated with kaempferol (1 μM) for varying time periods, and the cell lysates were subjected to Western blot analysis using antibodies specific for phospho-EGFR (Tyr1068), EGFR, phospho-Src (Tyr416), Src, phospho-AKT (Ser473), total AKT, phospho-ERK1/2 (Thr202/Tyr204), total ERK, and GAPDH. Relative pixel intensities for p-EGFR, p-Src, p-AKT, and p-ERK1/2 were measured by for p-EGFR/EGFR, p-Src/Src, p-AKT/AKT, and p-ERK1/2/ERK2 using Image J software. (B) Panc-1 cells were incubated with different doses of gefitinib for 60 min, and the cell lysates were subjected to Western blot analysis using antibodies specific for phospho-EGFR (Tyr1068), EGFR, phospho-Src (Tyr416), Src, phospho-AKT (Ser473), total AKT, phospho-ERK1/2 (Thr202/Tyr204), total ERK, and GAPDH. Relative pixel intensities for p-EGFR, p-Src, p-AKT, and p-ERK1/2 were measured by for p-EGFR/EGFR, p-Src/Src, p-AKT/AKT, and p-ERK1/2/ERK2 using Image J software. (C-D) Miapaca-2, Panc-1, and SNU-213 cells were incubated in the absence or presence of gefitinib (0.01 μM) for 1 h, and further treated with varying doses of kaempferol for 6 h. The migration activities were evaluated using the Transwell-migration assay (*p*-value by Student’s *t* test and are representative of three individual experiments, asterisks indicate a significant difference compared to gefitinib absence and presence, *p < 0.05, **p < 0.01).

We have previously shown that EGFR-related signaling pathway is of vital importance for migratory activity of pancreatic cancer cells [25]. In addition, mitogen-activated protein kinase (MAPK) signaling pathways are also critical for migration and invasion in various cancer cells including pancreatic cancers [25–27]. Especially, kaempferol induces apoptosis in two leukemia cell lines through the PI3K/AKT pathway [28]. Therefore, to understand the migratory inhibition signaling pathways of kaempferol treatment in human pancreatic cancer cells, we examined the phosphoylation level of EGFR, Src, AKT, and ERK1/2 after subjecting pancreatic cancer cells to time-dependent kaempferol treatments. Treatment of kaempferol, which was administered exogenously to pancreatic cancer cells, significantly decreased the phosphoylation levels of EGFR, Src, AKT, and ERK1/2 in a time-dependent manner in Miapaca-2 and Panc-1 cells (Fig 5A). In addition, inhibiting EGFR signaling pathway using pharmacological inhibitor, gefinitib suppressed Src, AKT, and ERK1/2 phosphoylation in a dose-dependent manner in Panc-1 cells (Fig 5B). Similar results were also obtained from Miapaca-2 cells (data not shown). We further verified the involvement of EGFR pathway in modification of signal transduction by kaempferol treatment. Dose-dependently treated with kaempferol (0, 0.2, 0.5, and 1 μM) significantly inhibited migration of Miapaca-2, Panc-1, and SNU-213 cells, which was effectively suppressed by pretreatment of sub-lethal dose of gefitinib (0.01 μM) in Miapaca-2, Panc-1, and SNU-213 cells, respectively (Fig 5C, 5D and 5E). These results indicated that kaempferol specifically inhibits the serum-induced migration through the blockade of EGFR related signaling pathway.

**Fig 6:**
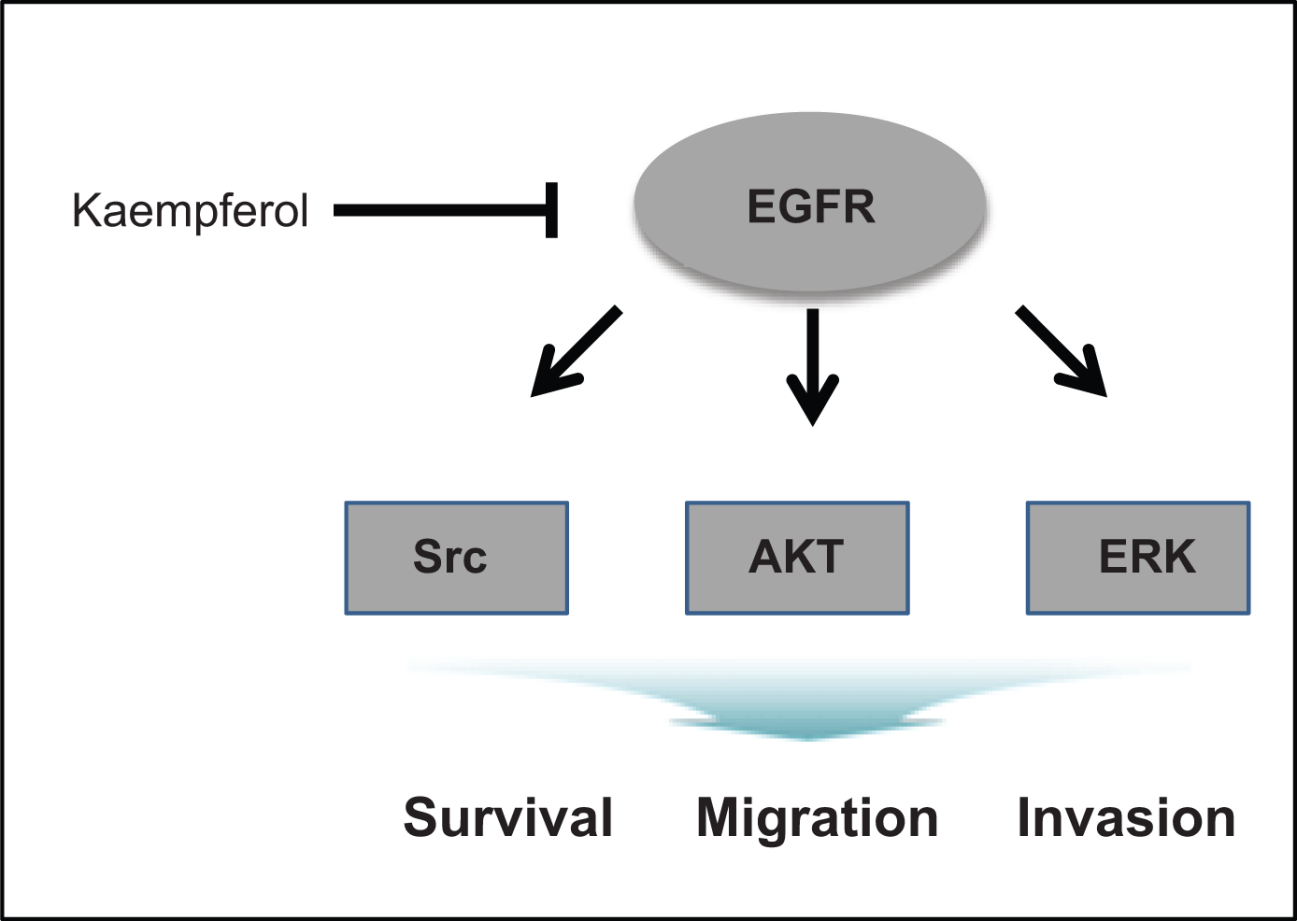
Kaempferol mechanism of action to inhibit growth and migration through the blockade of epidermal growth factor receptor-related signaling pathway in human pancreatic cancer cells

Collectively, these results clearly indicate that kaempferol efficiently inhibits the migratory activity by blocking the EGFR related Src, AKT, and ERK1/2 signaling pathway in human pancreatic cancer cells (Fig 6).

## Discussion

In this study, we determined that the migratory activity of human pancreatic cancer cells was significantly inhibited even at relatively low dosages of kaempferol treatment. We also certified that kaempferol effectively inhibits pancreatic cancer cell growth through the apoptosis *in vitro* in a dose-dependent manner. In addition, we selected 24 targets of the kaempferol using the TCMSP database, and ascertained that anti-cancer effects of kaempferol treatment in pancreatic cancer cells were caused by the blockade of EGFR related Src, AKT, ERK1/2 signaling pathway.

Although researchers have developed state-of-the-art diagnostic tools and comprehensive targeted therapies to inhibit pancreatic cancer, the overall survival rate of patients has not yet improved [27,28]. One of the critical features of pancreatic cancer is its aggressive migratory activity, which is responsible for the development of unrespectable, locally advanced, or metastatic pancreatic cancer [29]. In this report, kaempferol had significant anti-migratory effect in pancreatic cancers.

The anti-cancer effect of kaempferol on pancreatic cancer cell growth may be due to its anti-oxidation and cytotoxic activity [30,31]. In addition, kaempferol inhibits the activity of the following enzymes linked to cell growth and signaling pathway: cAMP-phosphodiesterase, tyrosine kinase, DNA topoisomerase II, topoisomerase I, and myosin light chain kinase [32–34]. Kaempferol treatment also inhibits fatty acid synthesis (FAS) and cell growth, and induces apoptosis in both prostate and breast cancer cells. This indicates that the induction of apoptosis in various cancer cells can be associated with the inhibition of FAS [35,36]. In agreement with previous studies, we observed that kaempferol inhibited cell growth by causing apoptosis of cancer cells through the decreased PCNA and cleaved caspase-3 pathway; this inhibition of cancer cells was achieved by administering kaempferol in different dosages. But, the inhibitory effect was diminished when the pancreatic cancer cells were subjected to pre-treatment of z-VAD-FMK. Zhang et al. reported the dramatic effect of *Ginkgo biloba* extract kaempferol on viability inhibition at low concentrations [9]. In our study, kaempferol showed moderate viability inhibition in excessive dosages of kaempferol in Miapaca-2 and Panc-1 cells. The differences between the studies may reflect the cell line conditions [37] or different source of kaempferol.

The chemical structure of the flavonoid has been correlated with its bio-activity [38–40]. Das et al. proved that bio-activity is affected by the structure of the flavonoid in refined palm oil. Flavonoid molecules with a hydroxyl group on A and B rings and the C3 position had a tendency of potent bio-activity [41]. In agreement, the most effective kaempferol has the potent polyhydroxyl groups on A, B, and C rings compared to kaempferol-3-O-glucoside and to kaempferol-4’-O-glucoside.

Kaempferol can block the development of metastatic cancer by inhibiting matrix metalloproteinase-3 activity in highly invasive to breast cancer cell line MDA-MB-231 [15]. Kaempferol treatment also inhibited the migration of MDA-MB-231 cells through the blockade of c-MET and AKT signaling pathways [14]. However, there are no previous reports about the effects of kaempferol treatment on migration of pancreatic cancer cells. Notably, kaempferol has the potential to inhibit effectively the migratory activity of human pancreatic cancer cells without any toxicity, even at relatively low dosages. In addition, high dosages of kaempferol (10 and 100 μM) more significantly inhibited the migration of pancreatic cancer cells. However, maximum dosages also significantly affected HUVEC migration.

Due to the remarkable development of bioinformatic-fields related to cancers, we could select 24 pancreatic cancer cell targets of kaempferol [42]. In particular, the fact that EGFR and hepatocyte growth factor receptor (HGFR) were targets in pancreatic cancers to kaempferol was most inspiring. Interestingly, there was no correlation between pancreatic cancers and kampferol derivatives, kaempferol-3-O-glucoside, and kaempferol-4’-O-glucoside in the TCMSP database. It might be the potential cause of inefficacious activities of kaempferol-3-O-glucoside and kaempferol-4’-O-glucoside in pancreatic cancer cell, however, further studies are needed to understand the fine mechanism about the distinction of kaempferol and its derivatives.

EGFR and HGFR are representative receptor tyrosine kinases that are critical causes of aggressively malignant pancreatic cancers [17,43,44]. Here, we verified that treatment of kaempferol effectively decreases the phosphoylation of EGFR, Src, AKT, and the ERK1/2 pathway. Gefinitib also inhibited the phosphoylation of EGFR, Src, AKT, and ERK1/2 pathway, and furthermore pretreatment of gefinitib diminished the effect of kaempferol on migration of pancreatic cancer cells. Thus, we validated the information from the TCMSP database that EGFR might be the target of kaempferol in pancreatic cancers. Additionally, the correlation between HGFR and kaempferol cannot be ignored. Precise study of the mechanism should be conducted to understand the anti-cancer effects of kaempferol in HGFR expressed pancreatic cancers.

In this study, we have shown that kaempferol could act as a potent inhibitor of pancreatic cancer cells migratory phenotype, functioning through the blockade of EGFR related Src, AKT, and ERK1/2 signaling pathway. Therefore, kaempferol is a protective and easily available anti-pancreatic cancer reagent that can inhibit the aggressive growth and frantic migratory activity of pancreatic cancers.

## Supporting Information

S1 Fig Sensitivities of kaempferol and its derivates in human pancratic cancer cells viability. Miapaca-2, Panc-1, and SNU-213 human pancreatic cancer cells were incubated in varying doses of kaempferol and its derivatives kaempferol-3-O-glucoside and kaempferol-4’-O-glucoside for 72 h. The viability was measured by WST-1 assay (data represent the percentage ± SD and are representative of three individual experiments, *p < 0.05, **p < 0.01).
(EPS)

S2 Fig The effect of different doses of kaempferol deriviates on migration of human pancreatic cancer cells. (A-C) Miapaca-2, Panc-1, and SNU-213 cells were incubated with varying doses of kaempferol-3-O-glucoside and kaempferol-4’-O-glucoside for 6 h. The migration activities were evaluated using the Transwell-migration assay (data represent the percentage ± SD and are representative of three individual experiments, *p < 0.05).
(EPS)
